# Clinical and Biochemical Data of Adult Thalassemia Major patients (TM) with Multiple Endocrine Complications (MEC) versus TM Patients with Normal Endocrine Functions: A long-term Retrospective Study (40 years) in a Tertiary Care Center in Italy

**DOI:** 10.4084/MJHID.2016.022

**Published:** 2016-04-12

**Authors:** Vincenzo De Sanctis, Heba Elsedfy, Ashraf T. Soliman, Ihab Zaki Elhakim, Christos Kattamis, Nada A. Soliman, Rania Elalaily

**Affiliations:** 1 Pediatric and Adolescent Outpatient Clinic, Quisisana Hospital, Ferrara, Italy; 2 Department of Pediatrics, Ain Shams University, Cairo, Egypt; 3 Department of Pediatrics, Division of Endocrinology, Alexandria University Children’s Hospital, Alexandria; 4 First Department of Paediatrics, University of Athens, Athens, Greece; 5 Ministry of Health, Alexandria, Egypt; 6 Department of Primary Health Care, Abu Nakhla Hospital, Doha, Qatar

## Abstract

**Introduction:**

It is well known that the older generation of adult TM patients has a higher incidence of morbidities and co-morbidities. At present, little information is available on adult TM patients with multiple endocrine complications (MEC). The main objectives of this longitudinal retrospective survey were: 1) to establish the incidence and progression of MEC (3 or more) in TM patients; 2) to compare the clinical, laboratory and imaging data to a sex and age-matched group of TM patients without MEC; 3) to assess the influence of iron overload represented by serum ferritin (peak and mean annual value at the last endocrine observation).

**Patients and methods:**

The study was started in January 1974 and was completed by the same physician at the end of December 2015. The registry database of the regularly followed TM patients from diagnosis included 145 adults (> 18 years). All TM patients were of Italian ethnic origin. Eleven out of 145 patients (7.5 %) developed MEC. Twenty-four other patients (12 females and 12 males) had a normal endocrine function (16.5 %) and served as controls.

**Results:**

In our survey, four important, relevant aspects emerged in the MEC group. These included the late age at the start of chelation therapy with desferrioxamine mesylate (DFO); the higher serum ferritin peak (8521.8 ± 5958.9 vs 3575.2 ± 1801.4 ng/ml); the upper proportion of splenectomized (81.8 % vs. 28.5%) patients and poor compliance registered mainly during the peripubertal and pubertal age (72.7 % vs.16.6 %) in TM patients developing MEC versus those without endocrine complications. Furthermore, a negative correlation was observed in all TM patients between LIC and final height (r: −0.424; p = 0.031).

**Conclusions:**

Our study supports the view that simultaneous involvement of more than one endocrine gland is not uncommon (7.5 %). It mainly occurred in TM patients who started chelation therapy with DFO late in life and who had irregular/poor compliance to treatment. Therefore, prevention of the endocrine complications through adopting early and regular chelation therapy appears mandatory for improving the quality of life and psychological outcome of these patients. When diagnosing and managing patients with MEC, it is of paramount importance that the multidisciplinary team have excellent knowledge relating to these complications. In ideal circumstances an endocrinologist with experience of TM will form part of the regular multidisciplinary team caring for such patients.

## Introduction

More than five decades ago, thalassemia major (TM) was fatal in the first decade of life.^1^ This poor prognosis changed since the survival rates started to increase progressively thanks to the implementation of continuous and significant improvement of diagnostic and therapeutic methods, consisting mainly of an intensive transfusion program combined with chelation therapy and imaging methods.^2^

Regular red blood cell (RBC) transfusions eliminate the complications of anemia, compensatory bone marrow expansion, bone changes and splenomegaly, restore the physiological growth throughout childhood and extend survival. The most serious disadvantage of life-saving transfusions is the inexorable accumulation of iron within tissues.^2^ Iron is physiologically stored intracellularly in the form of ferritin, a protein whose synthesis is induced upon the influx of iron. When the storage capacity of ferritin is exceeded, pathological quantities of metabolically active iron are released intracellularly in the form of hemosiderin and free iron within an expanded labile pool. This metabolically active iron catalyses the formation of free radicals, which damage membrane lipids and other macromolecules, leading to cell death and eventually organ failure.^3^

Other factors contributing to the variability of cellular iron overload are: a) the cell surface transferrin receptors and the capacity of the cells to deploy defence mechanisms against inorganic iron; b) individual susceptibility to iron toxic effect; c) the development of organ(s) damage secondary to persisting severe iron overload in the years preceding iron chelation therapy; and d) liver disorders, chronic hypoxia and associated endocrine complications, such as diabetes. 3

Recent advances in chelation therapy with new oral iron chelators and in imaging methods for assessing organs’ iron content resulted in striking improvements in outcomes for younger patients with TM, but few older patients have benefited from these improvements since the first years of life. Therefore, it is well known that the older generation of adult TM patients have higher morbidities and co-morbidities such as heart disease (heart failure and arrhythmias), chronic hepatitis (which may evolve into cirrhosis and rarely, in hepatocellular carcinoma), endocrine disorders (hypogonadism, hypothyroidism, diabetes, hypoparathyroidism), stunted growth and osteoporosis that limit the quality of their life. At present, little information is available for adult TM patients with multiple endocrine complications (MEC).^4^–^7^ Consequently, it was considered worthwhile to undertake a substantial study on the epidemiological and clinical issues on adult TM patients with MEC. The main objectives of this longitudinal retrospective survey were: 1) to explore the incidence and progression of MEC (3 or more) in TM patients; 2) to compare the clinical, laboratory and imaging data to sex and age matched TM patients without MEC; 3) to assess the influence of iron overload represented by serum ferritin levels (the highest level found during the follow-up and the value at last endocrine evaluation).

## Patients and Methods

### Setting, research design, and definitions

The study was started by VDS in January 1974 at the Pediatric and Adolescent Outpatient Clinic of Ferrara and was completed by the same physician at the end of December 2015 at the Quisisana Pediatric and Adolescent Outpatient Clinic of Ferrara.

Ethical approval for the study was obtained at the beginning of the study in accordance with local institutional requirements and in accordance with the Declaration of Helsinki (http://www.wma.net). All procedures were carried out with the adequate understanding and consent of parents or patients.

Inclusion criteria were: 1) patients with homozygous thalassemia (TM) based on hematological criteria; 2) adults with TM who were regularly followed in the same center (annually or bi-annually) and by the same physician from infancy to adulthood; 3) TM patients with and without MEC (3 or more endocrine complications).

β-TM was the term applied to patients who had either no effective production (as in homozygous β^0^ thalassemia) or severely limited production of β-globin. The diagnosis was confirmed by demonstrating thalassemia trait in both parents, lack of β-globin chain synthesis, absent HbA, a percentage of HbF of 95–98% and of HbA_2_ > 3,5%.

Exclusion criteria were: 1) non-transfusion-dependent thalassemias; 2) mental illness (depression, anxiety disorders, eating disorders and addictive behaviors); 3) renal insufficiency; 4) history of severe head trauma and brain injury; 5) alterations in nutritional status with significant loss of weight and/or the presence of depression; 6) bone marrow transplanted patients; 7) HIV positivity; 8) TM patients with incomplete data.

Data collected included: demographic characteristics, age at first transfusion, the interval between transfusions, compliance to iron chelation, anthropometry (weight, height, BMI), vital signs (blood pressure, heart rate, blood pressure) and pubertal status and associated endocrine complications.

Height and weight have been measured according to international recommendations.^8^,^9^ Body weight was measured, wearing minimal underclothes, to the nearest 100 g on properly calibrated scales. Short stature was defined as height below the third percentile on the 2006 Italian height chart. 10 Body mass index (BMI) was calculated (weight in Kg/height in m^2^). A subject was considered overweight when the BMI was between 25 and 30 and obese above 30.^11^

Delayed puberty in girls was defined as the absence of breast development by the age of 13 years; primary amenorrhea as the absence of menarche by the age of 16 years or a time gap of greater than 5 years between thelarche and menarche; secondary amenorrhea as the absence of menstruation for a period of 6 months at any time after menarche. Adolescents with delayed and arrested puberty were evaluated for pituitary–gonadal axis integrity.^11^

In boys, puberty was considered delayed if testicular growth (measured by a Prader orchidometer), was less than 4 ml by the age of 14 years.^11^ Hypogonadism (HH) and arrested puberty (AP) were defined as the absence of testicular enlargement (> 4 ml) by the age of 18 years or the lack of complete pubertal development for more than 5 years after the start of puberty. Adolescents with delayed and arrested puberty were evaluated for pituitary–gonadal axis integrity.^11^

All patients with insulin-dependent diabetes were monitored for the development of complications (urine albumin/creatinine ratio, renal function, fundus examination, blood pressure, foot examination and lipid profile).^12^ The metabolic control was assessed by home self-capillary blood glucose monitoring (SGMS) and periodic fructosamine estimation (fructosamine < 322 μmol/l was considered equivalent to HbA1c < 7.0%).^12^

Self-reported questionnaires (SRQs), patients or care providers interviews and random urinary iron excretion were used to assess the degree of compliance with chelation therapy as high (administering >90%), moderate (51–90%), poor (1%–50%) or non-compliant (0%). 13

### Blood sampling and analytical procedures

All blood samples were collected in the morning (08.00 – 09.00 am) after an overnight fast, and 1–2 weeks after blood transfusion.

Growth hormone (GH) stimulation test was required if:

- Height was below the 3^rd^ percentile or 2 SD below the mean height for age and sex.
- Height was within normal percentiles, but growth velocity (GV) was below the < 10^th^ percentile over 6–12 months.
- The patient was excessively short for his/her mid-parental height

In children and adolescents, a GH deficiency (GHD) was diagnosed if the peak GH values were <10 ng/mL in two pharmacological provocative agents (arginine, clonidine or glucagon were used as stimulants). In cases where a GH-releasing hormone (GHRH)/arginine test was performed, body mass index (BMI) dependent cut-offs were used. Severe GHD after arginine plus GHRH stimulation test was defined by GH peak <9 μg/l while partial GHD was defined as peak GH between 9 to 16.5 μg/l.^14^

Adolescents with delayed or arrested puberty were evaluated for pituitary–testicular/ovarian axis integrity (luteinizing hormone–LH and follicle stimulating hormone–FSH before and after stimulation with gonadotrophin releasing hormone (Gn-RH stimulation test), prolactin, estradiol in females and testosterone in males. Blood samples were assayed for FSH and LH before and 20, 40, 60 and 120 minutes after injection.

Other investigations included: a) thyroid function tests (free thyroxine-FT4 and thyrotropin-TSH), b) bone profile (calcium, phosphorus, albumin, alkaline phosphatase, PTH and bone densitometry, c) morning insulin-like growth factor 1(IGF-1), glucose and insulin in basal state and after oral glucose tolerance test (OGTT), and basal serum cortisol.

OGTT was first performed in 1975. Since 1981, the test has regularly been performed every 1–2 years in patients over the age of 11 years. The diagnosis of diabetes mellitus was based on World Health Organization (WHO) and American Diabetes Association (ADA) criteria.^15^,^16^

Hypoadrenalism was diagnosed if basal cortisol was 3.5 μg/dl (98 nmol/liter) or less.^17^ Thyroid dysfunctions were categorized as overt hypothyroidism (low FT4, increased TSH levels) and subclinical hypothyroidism (normal FT4 and increased TSH concentration: > 5 TSH mIU/ml). Central hypothyroidism was defined as an inappropriately low serum TSH concentration in the presence of subnormal serum FT4 concentrations.^11^,^18^ Anti-thyroid antibodies (ATA) were determined by anti-thyroglobulin (anti-Tg) and anti-thyroid peroxidase antibodies (anti-TPO) by commercially available immunoassay, in TM patients with primary hypothyroidism.

Hypoparathyroidism was diagnosed when there was low serum calcium concentration, increased serum phosphate and low serum parathyroid hormone (reference range 13–54 pg/ml), or a PTH level inappropriate for the calcium level.^11^,^19^ Hyperprolactinemia was defined as a basal level greater than the locally derived normal assay reference range.^20^

Serum FSH, LH, prolactin, estradiol, testosterone, FT4, TSH, PTH, and cortisol were measured by radioimmunoassay and chemiluminescent assay. Plasma total IGF-1 was measured by a chemiluminescent immunometric assay (CLIA) method (Nichols Institute Diagnostics, San Juan, CA).^21^ The sensitivity of the test was 6 ng/ml, whereas the intra and interassay coefficients of variation (CVs) of our in-house pooled serum control sample were 4.8% and 6.7%, respectively. The reported analytic sensitivity of this assay is 6 to 25 ng/ml (normal values set at the 2.5th–97.5th percentile were: 95.6–366.7 ng/ml for ages 25 to 39 yrs, 60.8–297.7 ng/ml for 40 to 59 yrs).^21^

Other parameters were determined using commercially available automated immunoassays. The intra- and interassay CV for all methods were < 5.8% and < 7.8%, respectively.

To evaluate liver functions, serum concentrations of alanine aminotransferase (ALT), gamma glutamyl transferase (γ GT), total and direct bilirubin, total proteins, albumin and international normalization ratio (INR) were measured. Urea, creatinine, and electrolytes were also measured. Screening assays for hepatitis C virus seropositivity (HCV ab and HCV-RNA) and virus genotype were performed applying appropriate laboratory methods.

### Assessment of iron overload

Iron overload was assessed by direct and indirect methods. At the beginning of the study, it was assessed only by measuring serum ferritin level. Iron overload was classified as mild (ferritin < 1000 ng/ml), moderate (ferritin >1000 ng/ml and < 2000 ng/ml) or severe (ferritin >2000 ng/ml).^22^

Serum ferritin was measured at the beginning by radioimmunoassay at a serum dilution of 1:1000 (normal values ± SD: males 108 ± 68 ng/ml, females 32 ± 25 ng/ml) and in the last years by immune, enzymatic and electrochemiluminescence immunoassays. The manufacturer’s normal reference range values were 30–350 μg/l in males and 15–150 μg/l in females.^23^

From 2005, in six out of eleven TM patients with MEC and 20/24 patients without endocrine complications, liver iron concentration (LIC) and cardiac T2* were assessed, by magnetic resonance imaging (MRI) using a 1.5 T scanner (GE Signa/Excite HD, Milwaukee, WI, USA) These were performed within the Myocardial Iron Overload in Thalassemia (MIOT) network, where MRI scans using homogeneous, standardized and validated procedures. 22, 24 A conservative cutoff value of heart T2* > 20 ms was considered normal. 24 Liver T2* values were converted into MRI liver iron content (LIC) values using the calibration curve introduced by Wood et al. 25 LIC values were expressed as mg/g dry weight (dw). 25 LIC (mg Fe/gr dw) were classified into mild (LIC > 3 and < 7), moderate (LIC > 7 and < 14) and severe overload (LIC > 14). 22

### Statistical analysis

Standard computer program SPSS for Windows, release 13.0 (SPSS Inc, Tulsa, IL, USA) was used for data entry and analysis. All numeric variables were expressed as mean ± standard deviation (SD). Comparison of different variables in the two groups was made using unpaired - student t-test and Mann-Whitney test for normal and nonparametric variables respectively. Chi-square (χ^2^) test was used to compare the frequency of qualitative variables among the different groups. Pearson’s and Spearman’s correlation tests were used to study correlations between variables with parametric and non-parametric distributions respectively. p < 0.05 was considered significant.

## Results

### Patients’ characteristics

**Figure 1:**
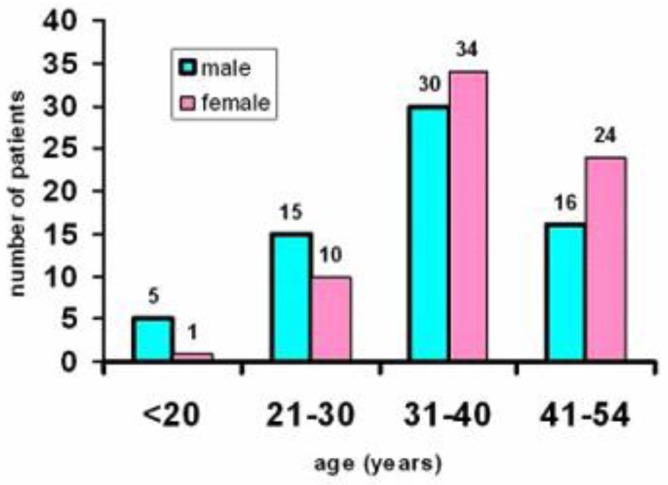
Age distribution of 145 thalassemia major patients regularly followed from diagnosis in the Endocrine Unit.

The registry database of the regularly followed TM patients from diagnosis included 145 adults (> 18 years; Figure 1). All TM patients were of Italian ethnic origin. Eleven out of 145 patients (7.5 %) developed MEC. Twenty-four (12 females and 12 males) had a normal endocrine function (16.5 %) and served as controls. One hundred and ten patients (77.2%) had either 1 to 2 endocrine complications or did not meet the inclusion criteria.

The baseline demographic, anthropometric and clinical data of the MEC and non-MEC groups are summarized in Table 1. The mean (± SD), age, standing height, weight, and BMI did not differ between TM patients with and without MEC. All patients without MEC had spontaneous and full pubertal development.

In the MEC group, two diabetic patients were obese, and one diabetic female was overweight.

Three female patients without MEC were classified as overweight, and none was obese. A male patient (38 years) with MEC had biopsy – proven hepatic cirrhosis. One female patient (40 years) with hypoparathyroidism, treated with 1,25(OH)D and oral calcium, had an associated diffuse cerebral calcifications in the deep white matter, posterior fossa, basal ganglia and both thalami on computed tomography scan.

### Transfusion management

Transfusion management of TM patients was changed over the time. Before 1972, blood transfusions were given when anemia was severe enough to cause symptoms. Thereafter, patients were regularly transfused every 2–3 weeks in order to maintain the mean hemoglobin (Hb) level at 9.5 g/dl (from 1972 to 1978), at 11.0 g/dl up to 1981, 12.5 g/dl till 1986 and 11 g/dl from 1986 till present. At the last endocrine examination, all TM patients were on regular transfusions (pre-transfusional Hb level 9 ± 0.3 g/dl (Table 1).

### Splenectomy

Patients were splenectomized when transfusion requirements of packed red cells increased to 180–220 ml/kg/yr and/or in the presence of other signs of hypersplenism such as leukopenia, thrombocytopenia or an enormous spleen. Seven TM patients (63.6%) with MEC had splenectomy at a mean age of 11 years (range: 5–25 years) and in 7 patients (29.1%) without MEC were splenectomized at a mean age of 14 years (range: 11–25 years) (p = 0.008)

### Evolution of chelation therapy and iron overload

Treatment with intramuscular desferrioxamine mesylate (DFO) at a dose of 20 mg/kg body weight (BW) was available for most patients since 1969. Regular subcutaneous (SC) DFO infusion was started in 1978 in patients older than 2 years. Initially, the recommended DFO dose was 20 mg/kg BW administered daily at night, by infusion pump over 10 hours. Based on transfusional iron input the dose increased to 40 mg/kg BW in 1982 and up to 60 mg/kg BW in 1984. Ascorbic acid was added orally at a dose of 2–5 mg/kg (maximum dose 200 mg) in a selected group of patients.

Since 1995, oral chelator deferiprone (DFP) has been available; it was given at a dose of 75 mg/kg BW to some patients over the age of 11 years. In the following years, combined therapy with daily DFP and subcutaneous DFO for 3–6 days/week was given to patients with severe iron overload and high iron input. In 2007, the new oral chelating agent deferasirox (DFX) was introduced at a dose of 25–30 mg/kg BW for patients in whom treatment with DFO was contraindicated or inadequate.

Chelation therapy in the two groups of patients at last evaluation is reported in table 1. Subcutaneous DFO infusion was started at a mean age of 11.9 years (range 3.4–18 years) in TM patients with MEC and at a mean age of 6.2 years in patients without MEC (range 2 – 14.4 years) (p = 0.001).

The mean serum ferritin levels reported in the two groups of TM patients are reported in table 2. The highest (peak) serum ferritin level found in the two groups was significantly greater in TM with MEC (8521.8 ± 5958.9 vs. 3575.2 ± 1801.4 ng/ml; p = <0.001); but not statistically different in the two groups at the last observation (Table 2).

Global cardiac T2* values expressed in msec were < 20 in 2/6 patients (33.3%) with MEC vs. 2/20 (10%) without MEC (p = NS). In the course of follow-up, LIC was assessed in 6/11 TM patients with MEC and 20/24 TM patients without MEC (Table 2). A LIC ≥ 14 mg/g dry weight was present in 3 patients without MEC and in none of the group with MEC (p = NS). These patients showed a significant improvement of LIC over time while their iron chelation therapy was being intensified with DFO plus DFP.

In general, the self-reported questionnaires and patients or care providers interviews for compliance to chelation therapy in the course of long-term follow-up was poor mainly during the peripubertal and pubertal age (< 18 years) in 8/11 (72.7%) TM patients with MEC and 4/24 (16.6 %) TM patients without MEC (p = 0.002; Table 1).

### Vaccinations, liver enzymes, and hepatitis C virus infection

Vaccinations against pneumococcus and haemophilus influenzae type B, hepatitis B, and A were available since 1992, 1983 and 1995, respectively*.* HIV and HCV antibodies have been tested annually since 1985 and 1991, respectively. All 35 patients enrolled in the study were tested for hepatitis C virus (HCV); 33 (94.2%) were HCV seropositive. HCV RNA positivity was present in 4/11 patients with MEC and 6/24 patients without MEC (Table 2). Two different HCV genotypes, 1b, and 2a were identified. Two HCV-RNA positive patients had been treated with interferon monotherapy and 2 with interferon and ribavirin. A sustained virologic response (SVR) was observed in two patients.

TM patients with MEC had higher ALT and serum γGT concentrations that were not statistically different compared to controls (non- MEC group). The MEC group had significantly higher ALP levels versus the controls (Table 2).

### Growth and endocrine complications

Short stature was present in 9 out of 35 patients (3 males). Short stature was secondary to: a) GH deficiency (GHD) in 2 patients (22.2 %) who developed MEC; b) DFO “toxicity” causing marked platyspondilosis (4 patients without MEC; 44.4 %); c) familial short stature (1 patient with MEC; 11.1 %); d) constitutional short stature (1 patient without MEC; 11.1 %) and e) severe chronic liver diseases (1 patient with MEC; 11.1%). In general, a negative correlation was observed in all TM patients between LIC and final height (r: −0.424; p = 0.031) (Table 3).

IGF-1 levels were below -2SD in all TM patients with MEC compared to the percentile of healthy subjects. 21 One patient with GHD refused treatment, and one was treated during peripubertal age with conventional doses of rhGH. After the first 12 months of treatment, rhGH was suspended because of poor response (< 2 cm increment above the basal value). GH secretion was reassessed in both patients in young adult life. Persisting severe GHD was observed in one patient and a partial GHD (GH peak: 9.2 ng/ml) in the second patient.^14^,^26^,^27^ Both developed MEC.

Eleven patients (3 males) developed insulin-dependent diabetes mellitus at a mean age of 22.5 years (range 12–35 years). Eight patients (3 males) presented with subclinical hypothyroidism at a mean age of 20.2 years (range 12–32 years). One female patient (9%) developed central hypothyroidism at the age of 36 years and two females developed overt hypothyroidism (18.1%) at the age of 15 and 26, respectively. Secondary amenorrhea was registered in 3 patients at 34, 37 and 38 years, respectively.

The first and most endocrine complication was hypogonadotropic hypogonadism (36.3%) and insulin-dependent diabetes mellitus (36.3%) followed by hypothyroidism (18.1%) and hypoparathyroidism (18.1%). Secondary amenorrhea (27.2%) was the last observed complication in the MEC group.

No cases of autoimmune thyroiditis, hypoadrenalism, primary hypogonadism or hyperprolactinemia were observed.

Fifty-four percent of patients with MEC were receiving irregular hormone replacement therapy for hypogonadism or secondary amenorrhea. In the remaining patients, treatment was refused after discussing with every subject the pros and cons of sex steroid replacement therapy.

All patients with primary or central hypothyroidism were receiving levothyroxine, those with hypoparathyroidism calcium and calcitriol and the diabetic patients insulin. None of the diabetic patients was on antihypertensive medication or lipid-lowering agents (statins or fibrates). Two patients (20 %) had persistent microalbuminuria, and one (10 %) was diagnosed with non-proliferative diabetic retinopathy (NPDR). Two diabetic female patients aged 37 and 40 years had a global heart T2* value of 7 and 13.6 msec and a LIC value of 5 and 4.3 mg Fe/g dry wt, respectively. No other diabetic complication was documented.

## Discussion

In adult TM patients in this study, the prevalence of MEC was 7.5 %. A higher percentage was reported by Perera et al.^28^ in a retrospective cohort analysis of TM patients attending an ambulatory transfusion clinic. All their patients had, at least, one endocrinopathy, and 16 patients (55%) had three or more (≥3). Hypogonadism was the most prevalent followed by growth failure (less than 3rd centile) with a frequency of 55% and 35%, respectively.

A literature review of 593 TM patients showed a prevalence of MEC that varied between 1% to 10%. Those with more endocrinopathies (≥3) had a longer duration of transfusion therapy compared with those with fewer endocrinopathies. 5,29,30

The first and the most frequent endocrine complications diagnosed in our study were hypogonadotropic hypogonadism and insulin-dependent diabetes mellitus; diagnosed at a mean age of 18 and 22.5 years, followed by hypothyroidism and hypoparathyroidism (at a mean age of 20.2 and 21.7 years).

Although serum ferritin is used as index to start chelation therapy, it is not a very accurate indicator of total body iron burden as its level may be influenced by other factors, such as inflammation, liver damage, and vitamin C deficiency. However, a group of researchers has earlier shown that a high serum ferritin level during puberty (> 2500 ng/ml) is a risk factor for hypogonadism and a serum ferritin level of >3000 ng/ml, during the first decade of life is a predictor of short adult stature.^31^

In another study, TM patients with a serum ferritin level >2,500 μg/l, but not >1,000–2,500 ng/ml, were 3.53 times (95% CI 1.09–11.40) more likely to have diabetes mellitus, 3.25 times (95% CI 1.07–10.90) to have hypothyroidism, 3.27 times (95% CI 1.27–8.39) to have hypoparathyroidism and 2.75 times (95% CI 1.38–5.49) to have hypogonadism compared to patients with a serum ferritin level ≤ 1,000 ng/ml 32

In our survey four important, relevant aspects emerged in the MEC group. These are: the delay in initiation of chelation therapy with DFO; the significantly higher peak in TM with MEC group vs the non-MEC group (serum ferritin peak 8521.8 ± 5958.9 vs 3575.2± 1801.4 ng/ml); the higher percentage of splenectomized (81.8 % vs. 28.5%) patients and the poor compliance registered mainly during the peripubertal and pubertal age (72.7 % vs.16.6 %) in TM patients developing MEC versus those without endocrine complications (Table 1)

The spleen is a major constituent of the total body iron load in TMf patients and a rapid rise in serum ferritin level has been documented following the splenectomy in patients with hemoglobin H Constant Spring disease and TM patients.^33^,^34^ After splenectomy, the total body iron storage capacity decreased, whereas serum ferritin (p = 0.0085) and iron concentration in other organs appeared to increase despite the reduction in the rate of transfusions (p = 0.0001) and maintenance of hemoglobin levels. Normalization of the body iron stores at an early age could maintain the spleen at near normal capacity and avoid other complications (cardiac and hepatic).^33^,^34^

Although the compliance to chelation in our study was not fully portrayed, a better compliance during peripubertal and pubertal age (< 18 years) to treatment was associated with a better outcome. Non-compliance with therapy is a big threat to effective treatment and one of the most common problems encountered in clinical practice. We did not assess the motivation of patients to comply with chelation therapy. However, our personal experience showed that compliance of the TM patients was influenced by several factors, such as age, socio-economic status, lack of family support, lack of knowledge about the disease, concern or fear from side effects, chronicity, severity of the disease, lack of immediate benefit and presence or absence of complications and poor doctor-patient relationship. Furthermore, multiple drug therapy and complex treatments that interfered with daily life were also reasons for non-compliance.

Vullo and Di Palma, working for several years in the same Pediatric and Adolescent Outpatient Clinic of Ferrara, reported that compliance with chelation therapy also had a significant positive correlation with participation in supportive group conferences, parents educational level and high socioeconomic status. This finding supports the notion that health care workers must look beyond the individual when examining non-compliant behavior and also direct attention to the external factors, such as family dynamics and socioeconomic status.^35^

The existence of many methods to evaluate compliance with therapy reflects the absence of standard “gold standard” method. Indirect information regarding compliance with therapy is gathered through history taking, counting pills and using a patient’s diaries. However, it is well known that the information reported by patients, either verbally or in writing, is unreliable due to either inability to remember or false reporting in order to please or to avoid disapproval of the physician. 36 Nevertheless, the National Institute for Health and Care Excellence (NICE) guidelines have identified that whilst other types of measures are useful, self-report is an appropriate tool for clinical practice^37^, and recent reviews have shown that self-report has a moderate correlation with electronic monitoring.^38^,^39^ These data suggest that self-reported questionnaires (SRQs) can give a good estimate of medication adherence.

Our study has some limitations that should be mentioned. It was a single-centre study, and the enrolled number of TM patients with MEC was small. A larger study population could make the results more reliable. Furthermore, in our study, the peak serum ferritin level seems to be a good indicator for the development of MEC. However, it is well known that serum ferritin increases in the presence of associated acute and chronic disorders particularly inflammatory and hepatic conditions, such as chronic hepatitis, and, therefore, may limit the validity and effectiveness of ferritin as a predictive factor of endocrine dysfunction.

Also, the GH-IGF1 axis and the hypothalamic-pituitary-adrenal axis were not fully investigated in the current study. Although these limitations are important, it is unlikely to have had a significant effect on the validity of our findings.

## Conclusions

Epidemiological and clinical information about the development of MEC in patients with thalassemia as well as factors influencing its progression is still limited. Conflicting data are available in the literature on the use of serum ferritin level as a useful marker for endocrine dysfunction.

Our study supports the view that simultaneous involvement of more than one endocrine gland is not uncommon (7.5 %) in adult TM cohorts. It mainly occurred in TM patients who started chelation therapy with DFO late in life and had irregular/poor compliance to treatment. Therefore, continuous diligent treatment is the key to the management of thalassemia. We believe that our data could be replicated in developing countries where due to economic circumstances, inadequate transfusions and chelation therapy are a rule rather than the exception.^40^,^41^

In our study serum, ferritin levels at the final estimation did not statistically differ between patients with MEC and those without MEC denoting that late adhesion to therapy did not reverse endocrine complications. This supports the notion that poor compliance and poor chelation therapy can cause significant and irreversible tissue damage in many organs including endocrine glands. Therefore, prevention of the endocrine complications through adopting early and regular chelation therapy appears mandatory for the improving the quality of life and psychological outcome of these patients. Today many patients can benefit from modern treatment

Monitoring compliance is essential in such conditions since the final result is likely to be influenced by the adherence to the therapeutic regimen. Timely diagnosis and treatment of these disorders are often delayed as a result of focusing all attention on the primary hematological problems. In fact, endocrine complications represent a challenge that extends well beyond the hematology spectrum and requires close collaboration between many other clinical and research disciplines. Moreover, the simultaneous involvement of the liver and other organs makes the management of endocrine complications harder than usual.

Much of the morbidity and mortality from these complications can be reduced with regular surveillance, early treatment, and follow-up in a specialized multidisciplinary setting. We recommend that patients with MEC must be followed meticulously because of the possibility of the development of new complications, such as diabetic microvascular disease, cardiac, and hepatic diseases.

## Figures and Tables

**Table 1:** Clinical, laboratory data, compliance, and treatment in thalassemia major patients with and without multiple endocrine complications (MEC)

Variables	Thalassemic patients with multiple endocrine complications (mean ± SD) (11 patients)	Thalassemic patients without endocrine complications (mean ± SD) (24 patients)	P value

**Age (yr)**	35.2±4.3	35.9±4.0	0.642

**Sex (M/F)**	3/8	12/12	0.207

**Weight (Kg)**	61.3±11.5	55.8±8.1	0.173

**Short stature (< 3^rd^** **centile)**	3/11	6/24	0.6
**Males**	0/3	3/12	0.48
**Females**	3/8	3/12	0.46

**Body Mass Index (kg/m^2^)**	24.5±6.4	29.6±34.3	0.713

**Splenectomy (n)**	9/11	8/24	0.008

**Hypogonadotropic hypogonadism (n)**	7/11	-	NA
**Arrested puberty (n)**	1/11 (female)	-	NA
**Secondary amenorrhea (n)**	3/11	-	NA

**Diabetes insulin dependent (n)**	10/11	-	NA

**Hypoparathyroidism (n)**	4/11	-	NA

**Subclinical hypothyroidism (n)**	8/11		NA
**Overt hypothyroidism (n)**	2/11		NA
**Central hypothyroidism (n)**	1/11		NA

**Hypoadrenalism (n)**	0/11	-	NA

**Hyperprolactinemia (n)**	0/11	-	NA

**Chelation therapy**			
**DFO (n)**	5/11	11/24	
**DFP (n)**	2/11	4/24	0.953
**DFO + DFP (n)**	2/11	3/24	
**DFX (n)**	2/11	6/24	

**Poor compliance to chelation therapy (n)**	8/11	4/24	0.002

Legend: MEC –Multiple Endocrine Complications; NA: Not Applicable; deferoxamine (DFO), deferiprone (DFP), deferasirox (DFX)

**Table 2:** Relevant laboratory parameters and imaging data in thalassemia major patients with and without multiple endocrine complications (MEC)

Variables	Thalassemic patients with MEC (mean ± SD) (11 patients)	Thalassemic patients without MEC (mean ± SD) (24 patients)	P value

Global Heart T2* (msec)	30.7± 17.75	33.3± 10.66	0.748
**95% confidence interval**			
**Lower bound**	12.11	28.32	
**Upper bound**	49.38	38.31	

**Left ventricular ejection fraction (%)**	65.9± 3.1	65.5± 5.2	0.838

**Liver iron concentration (mg Fe/g dry wt)**	2.13± 2.25	5.7± 5.3	0.062

**ALT (U/L)**	58.3±59.4	41.0±37.5	0.540

**γ-GT (U/L)**	35.0±23.6	24.4±18.8	0.092

**Alkaline phosphatase (IU/L)**	254.5± 129.3	210.5± 167.1	0.016

**IGF1 (ng/ml)**	35.9±21.2	86.9±31.3	<0.001
**95% confidence interval for mean**			
**Lower bound**	21.7	73.715	
**Upper bound**	50.2	100.2	

**HCV-ab positive (n)**	11/11	22/24	0.324
**HCV –RNA positive (n)**	4/11	6/24	0.730

**Serum ferritin (ng/ml)**	1277.0±799.6	1474.0±1505.3	0.662
**95% confidence interval**			
**Lower bound**	739.87	838.43	
**Upper bound**	1814.32	2109.73	
**Peak serum ferritin (ng/ml)**			<0.001
**95% confidence interval**	8521.8± 5958.9	3575.2± 1801.4	
**Lower bound**	4518.56	2814.62	
**Upper bound**	12525.08	4335.96	
**Serum ferritin level > 2000 ng/ml at last observation (n)**	1/11	5/24	0.37

*Legend: MEC –Multiple Endocrine Complications.* Alanine Aminotransferase (ALT)= 5–40 U/L; γ Glutamyl transferase= 10–49 U/L; Alkaline phosphatase: 25–90 IU/L; INR= 0.9–1.2; *Normal values: IGF-1= 95.6–366.7 ng/ml for ages 25 to 39 yrs, 60.8–297.7 ng/ml for 40 to 59 yrs;* Serum ferritin= 30–350 μg/l in males and 15–150 μg/l in females.

**Table 3:** Statistically significant correlations between different variables in thalassemia major (TM) patients with and without multiple endocrine complications (MEC)

	r	p

**Variables in TM patients with MEC**		

ALT vs. total protein	0.431	0.035
	0.230	0.496

Peak serum ferritin vs. last serum ferritin	0.706	0.015

**Variables in TM patients without MEC**		

Age vs. last serum ferritin	−0.571	0.004

Weight vs. LEF (%)	−0444	0.038

Last serum ferritin and LIC	0.482	0.032

**All TM patients**		

ALT vs. total protein	0.383	0.023

LIC vs. height	−0.424	0.031

***Legend:*** Alanine Aminotransferase (ALT); Left Ejection Fraction (LEF); Liver Iron Concentration (LIC)
